# 

**Published:** 1879-10

**Authors:** 


### BOOKS AND PAMPHLETS RECEIVED.

The Nature and Treatment of Rabies or Hydrophobia. By F. M. Dolan, L. R. C. P. E. Being the report made for the *Medical Press and Circular*. Second edition; pp. 287, cl.; 1879. London: Ballière, Tindall & Cox. Chicago: Jansen, McClurg & Co.

Sexual Neurosis. By J. T. Kent, A. M., M. D. Pp. 144, cl.; 1879. St. Louis: Maynard & Bedford.

Physiology and Histology of the Cerebral Convolutions: Also Poisons of the Intellect. By C. Richet, A. M., M. D., PH. D. Translated by E. P. Fowler. Cl., pp. 170. New York: W. Wood & Co.

Medical and Surgical History of the War of the Rebellion. Part II, vol. 1, med. vol. Prepared under the direction of J. K. Barnes, Surgeon General of U. S. Army, by J. J. Woodward, Surgeon U. S. Army. Cl., pp. 869. Washington Government Printing Offices, 1879.

Transactions of the Thirty-Fourth Annual Meeting of the Ohio State Medical Society. Held at Dayton, June, 1879.

First Steps in Chemical Principles. By Henry Leffman, M. D. Cl., pp. 51; 1879. Philadelphia: Ed. Stern & Co.

American Cyclopædia of Domestic Medicine and Household Surgery. Illustrated. Edited by Sam. Payne Ford, M. D. Leather, pp. 1215. Chicago: E. P. Kingsley.

Transactions of the Medical Society of the State of Tennessee at its Forty-Sixth Annual Meeting, 1879.

Transactions of the Medical Association of Georgia. Thirteenth Annual Session at Rome, 1879. Atlanta, Georgia.

Reports of the St. Louis Medical Society on Yellow Fever. By W. Hudson Ford, A. M., M. D. Cl., pp. 320; 1879. St. Geo., O.; Rumbold & Co.

Annual Reports of the Supervising Surgeon General of the Marine Hospital Service of the United States, for the Fiscal Years 1876 and 1877. By J. M. Woodworth, M. D.

Report on Milk and Dairies in the City of New Orleans. Presented to the New Orleans Medical and Surgical Association, and unanimously accepted July 5, 1879.

The Metric System. By J. F. Baldwin, M. D. Read before the Ohio State Medical Society.

Remarks on Ovariotomy, with Relations of Cases and Peculiarities in Treatment. By Nathan Bozeman, M. D. Reprint from *Medical Record*, July and August, 1879.

Excision of Knee Joint with Cases. By Joseph Eastman, M. D. Reprint from Transactions of Indiana State Medical Society, 1879.

Reflex Cerebral Hyperæmia. By C. H. Hughes, M. D. Read before St. Louis Medical Society. Reprint from St. Louis *Medical and Surgical Journal*, June, 1879.

Atlas of Histology. By E. Klein, M. D., F. R. S., and E. N. Smith, L. R. C. P., etc. Parts V. and VI. Philadelphia: J. B. Lippincott & Co. Chicago: W. T. Keener.

The Yellow Fever Germ on Coast and Inland. A Discussion of Ship and Railroad Quarantine. Read before the Medical Association of Georgia. By H. F. Campbell, M. D. Reprint from Transactions.

Vegetarianism the Radical Cure for Intemperance. By H. P. Fowler. New York: M. L. Holbrook & Co.

Microscopical Studies on Abcess of the Liver. By J. C. Davis, M. D. Reprint from the *Archives of Medicine*, August, 1879.

A New Operation for Entropion and Trichiasis. By F. C. Hotz M. D. Reprint from *Archives of Opthalmology*. Vol. VIII., No. 2.

Sundry Facts, Opinions and Suggestions Derived from the Observation of Forty Cases of Tapeworm. By J. Bartlett, M. D. Reprint from Chicago MEDICAL JOURNAL AND EXAMINER, September, 1879.

---

DEFORMITIES OF MEDICAL JOURNALS.—We are pleased to find in a late issue of the *Pacific Medical and Surgical Journal*, some unfavorable comments upon the prevailing practice of inserting advertisements in the body of a medical journal. In this day, when so many sheets are merely subsidized organs of wholesale drug houses, the practice is common enough, and evidently intended to secure that sustenance at the hands of the drug man, which can not be obtained at the hands of the profession. The opinion seems to be prevalent that medical gentlemen are so stupid that they can not detect the weakness of these sheets when the evidence of it is carried on the face. Such an opinion may be well founded, but, we think differently. Enjoying, as we do, the pages of those of our exchanges which do not exhibit this defilement, we propose to keep our own equally clean.

### ANNOUNCEMENTS FOR THE MONTH.

### SOCIETY MEETINGS.

Chicago Medical Society—Mondays, Oct. 6 and 20.

West Chicago Medical Society—Mondays, Oct. 13 and 27.

### CLINICS.

MONDAY.

Eye and Ear Infirmary—2 p. m., Ophthalmological, by Prof. Holmes ; 3 p. m., Otological, by Prof. Jones.

Mercy Hospital—1:30 p. m., Surgical, by Prof. Andrews.

Rush Medical College—2 p. m., Dermatological and Venereal, by Prof. Hyde ; 3 p. m., Medical, by Dr. Bridge.

Woman's Medical College—2 p. m., Dermatological, by Dr. Maynard.

TUESDAY.

Cook County Hospital—2 to 4 p. m., Medical and Surgical Clinics.

Mercy Hospital—1:30 p. m., Medical, by Prof. Hollister.

WEDNESDAY.

Chicago Medical College—1:30 p. m., Eye and Ear, by Prof. Jones.

Rush Medical College—3:30 to 4:30 p. m., Diseases of the Chest, by Dr. E. Fletcher Ingals.

THURSDAY.

Chicago Medical College—1:30 p. m., Medical, by Prof. Quine.

Rush Medical College—3 p. m., Diseases of the Nervous System, by Prof. Lyman.

Eye and Ear Infirmary—2 p. m., Ophthalmological, by Dr. Hotz.

FRIDAY.

Cook County Hospital—2 to 4 p. m., Medical and Surgical Clinics.

Mercy Hospital—1:30 p. m., Medical, by Prof. Davis.

SATURDAY.

Rush Medical College—2 p. m., Surgical, by Prof. Gunn.

Chicago Medical College—2 p. m., Surgical, by Prof. Isham; 3 p. m., Neurological, by Prof. Jewell.

Woman's Medical College—11 a. m., Ophthalmological by, Dr. Montgomery.

Daily Clinics, from 2 to 4 p. m., at the Central Free Dispensary, and at the South Side Dispensary.

